# Classification of Approximal Caries in Bitewing Radiographs Using Convolutional Neural Networks

**DOI:** 10.3390/s21155192

**Published:** 2021-07-31

**Authors:** Maira Moran, Marcelo Faria, Gilson Giraldi, Luciana Bastos, Larissa Oliveira, Aura Conci

**Affiliations:** 1 Policlínica Piquet Carneiro, Universidade do Estado do Rio de Janeiro, Rio de Janeiro 20950-003, Brazil; mfaria@uerj.br (M.F.); odonto@uerj.br (L.B.); loliveira@uerj.br (L.O.); 2 Instituto de Computação, Universidade Federal Fluminense, Niterói 24210-310, Brazil; 3 Faculdade de Odontologia, Universidade Federal do Rio de Janeiro, Rio de Janeiro 21941-617, Brazil; 4 Laboratório Nacional de Computação Científica, Petrópolis 25651-076, Brazil; gilson@lncc.br

**Keywords:** bitewing radiography, neural networks, artificial intelligence, caries, dental radiography, diagnosis, dentistry

## Abstract

Dental caries is an extremely common problem in dentistry that affects a significant part of the population. Approximal caries are especially difficult to identify because their position makes clinical analysis difficult. Radiographic evaluation—more specifically, bitewing images—are mostly used in such cases. However, incorrect interpretations may interfere with the diagnostic process. To aid dentists in caries evaluation, computational methods and tools can be used. In this work, we propose a new method that combines image processing techniques and convolutional neural networks to identify approximal dental caries in bitewing radiographic images and classify them according to lesion severity. For this study, we acquired 112 bitewing radiographs. From these exams, we extracted individual tooth images from each exam, applied a data augmentation process, and used the resulting images to train CNN classification models. The tooth images were previously labeled by experts to denote the defined classes. We evaluated classification models based on the Inception and ResNet architectures using three different learning rates: 0.1, 0.01, and 0.001. The training process included 2000 iterations, and the best results were achieved by the Inception model with a 0.001 learning rate, whose accuracy on the test set was 73.3%. The results can be considered promising and suggest that the proposed method could be used to assist dentists in the evaluation of bitewing images, and the definition of lesion severity and appropriate treatments.

## 1. Introduction

**Figure 1:**
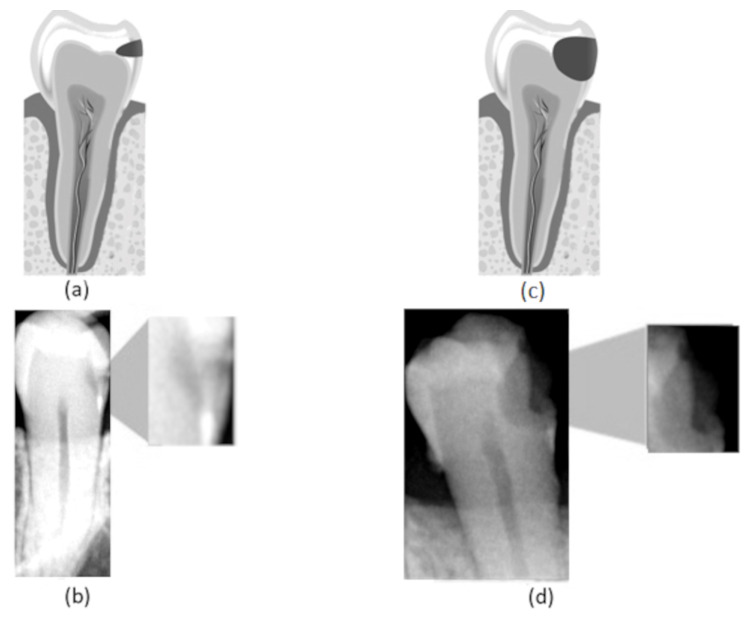
(**a**) Representation of an incipient caries lesion, (**b**) bitewing image with incipient lesion highlighted, (**c**) representation of an advanced caries lesion, (**d**) bitewing image with advanced lesion highlighted.

The high incidence of caries lesions suggests the importance of developing clinical decision support systems that aid specialists in evaluating such lesions. It is widely known that early diagnosis is crucial to initiate effective treatments for most diseases, increasing the chance of success. This is also applicable for oral diseases, including dental caries. Nevertheless, due to the lack of early diagnosis, caries lesions are frequently detected in advanced stages (Figure 1d), in which restoration is the only effective treatment [1]. This is especially harmful in cases in which these restorative treatments demand general anesthesia, as for children and special needs patients [2,3], since the use of anesthesia increases the patient risk. The impact of the use of anesthesia is not only perceptible in clinical routines but also in computational simulations [4]. All these facts confirm the urgency of the early detection of this kind of lesion, which is so important that even during the COVID-19 pandemic, several dentists decided to assess cases of pulp-related problems in their physical offices [5]. Along with the early diagnosis, the correct definition of the lesion’s stage is essential for assertive treatment planning.

**Figure 2:**
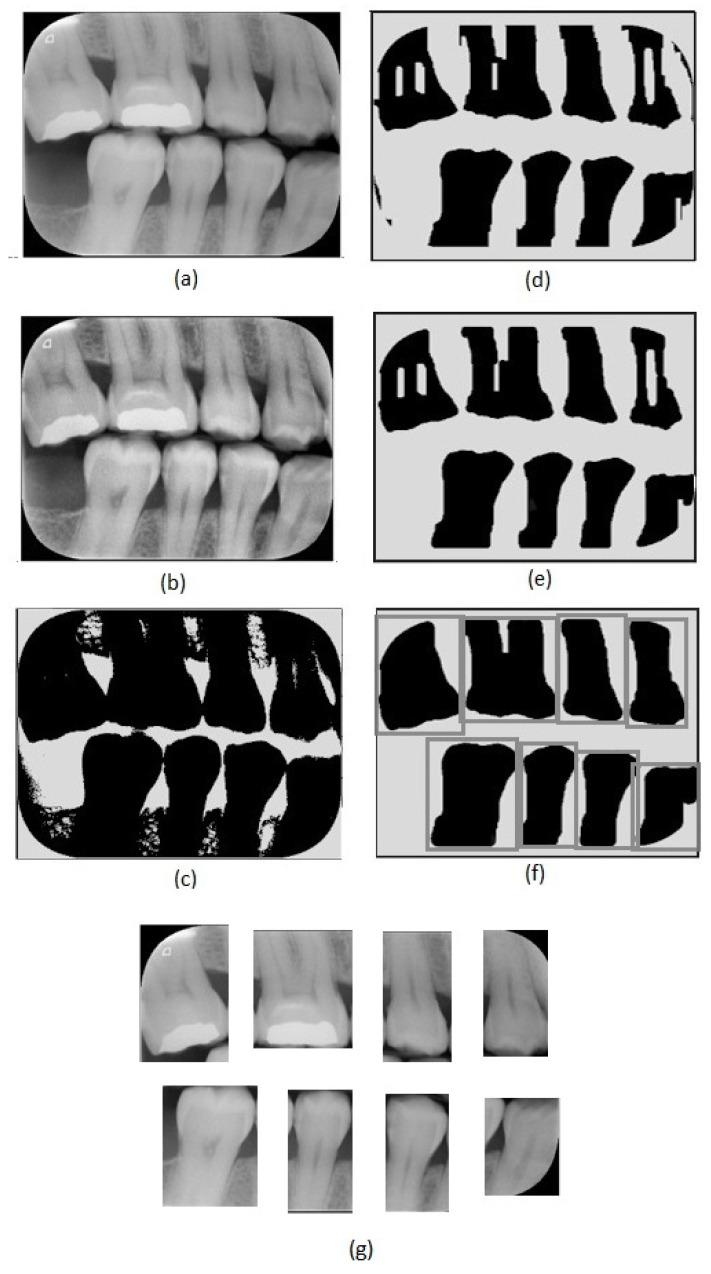
Image preprocessing steps: (**a**) original image, (**b**) adaptive histogram equalization, (**c**) Otsu’s thresholding, (**d**) erosion, (**e**) closing, (**f**) dilation and tooth region definition, (**g**) tooth images obtained.

The diagnosis of carious lesions usually includes not only clinical examination but also radiographic interpretation. The diagnosis of approximal caries is performed through image examinations, especially bitewing radiographs (Figure 2a), due to the localization of such lesions, which prevents clinical identification. Bitewing radiographs provide a more restricted and specific view, allowing a better evaluation of approximal lesions. They generally cover the area defined by the distal surface of the canine teeth to the distal surface of the farthest molar. The visual presentation of dental caries in bitewing radiographs consists of a dark area due to their low X-ray absorption [6]. A wide range of different phenomena may affect bitewing radiographies, such as cervical burnout [7], which can be difficult to detect. For that reason, the use of complementary tools based on computational techniques can help to achieve more robust evaluations.

In the last years, solutions based on Artificial Intelligence (AI) algorithms, especially deep-learning ones, have emerged in a wide range of application fields, demonstrating outstanding results. This trend is also perceptible in Dental Medicine [8,9,10,11,12,13,14,15]. Considering the analysis of radiographs as a complementary tool for diagnosis, the use of Convolutional Neural Networks (CNNs) to aid in the identification of several lesions has shown promising results [9]. CNNs consist of a specialized kind of machine learning algorithm for processing data that present a gridlike topology, such as images [16].

Recently, CNNs have been widely employed in the Cariology field [9]. In the work presented by Karimian et al. [13], a CNN was used to provide early detection of dental caries by processing optical coherence tomography (OCT) images of oral tissues to determine variations related to demineralization processes. Lee et al. [14] proposed the use of CNNs to detect dental caries on periapical radiographs. The networks implemented were based on the Inception architecture. In that work, the authors implemented three different models for caries identification in different tooth types: a premolar model, a molar model, and a final model for both premolar and molars. These models achieved impressive accuracy results (89.0%, 88.0%, and 82.0%, respectively). Choi et al. [17] proposed a system for automatic detection of proximal dental caries in periapical images, composed of four modules: horizontal alignment of teeth, probability map generation, crown extraction, and refinement. The proposed solution included a fully convolutional neural network. In the study presented by Sornan et al. [15], the authors proposed an algorithm based on artificial intelligence (logit-based artificial bee colony optimization), which analyzes gray-level co-occurrence matrix (GLCM) features of bitewing radiographs in order to identify dental caries; this method achieved an accuracy of 99.16%. Srivastava et al. [18] developed a Computer-Aided Diagnosis system to detect dental caries on bitewing radiographs based on a deep, fully convolutional neural network composed of more then 100 layers. When compared with human dentists, the system overcame their average performances in recall (80.5%), precision (61.5%), and F1-Score (70.0%).

Previous works that include AI algorithms for caries detection have not consider the lesion stage in their analysis. In this work, we developed a method to extract regions of interest referent to teeth in bitewing radiographs and classify them according to the severity of their approximal caries. The proposed method is based on image processing techniques and CNNs. We evaluated the performance of CNN models based on two widely used CNN architectures: Inception [19] and ResNet [20].

In this work, we consider three different caries stages based on their lesion severity: normal, incipient, and advanced. The normal class consists of teeth with no lesion. The incipient class denotes teeth with superficial lesions affecting the enamel—Figure 1a,b. Finally, the advanced class refers to teeth with advanced lesions, affecting a considerable part of the tooth, expanding into the dentin and the pulp—Figure 1c,d.

## 2. Materials and Methods

### 2.1. Datasets

This study included 480 different regions of interest referent to different teeth. These regions of interest were extracted from 112 bitewing radiographs. The bitewing examinations were obtained using a Sirona Heliodent Plus oral X-ray unit (Kavo Brasil Focus), and were archived as grayscale digital images in the JPEG format. We used the parameters recommended by the manufacturer for digital image capture: 70 kV and 7 ma. The only variation was the exposure time, ranging from 0.25 to 0.64 s, according to the patient’s physical type. In addition, the EXPRESS™ Origo imaging plate system (Intraoral imaging plate system; https://www.kavo.com/dental-xray-machines-diagnostics/intraoral-x-ray, accessed on 19 February 2021) by KaVo Dental (Biberach an der Riss, Germany) was used in the image capture. The acquisition process was performed at Policlínica Piquet Carneiro, an Institute associated with the State University of Rio de Janeiro.

For this study, we defined the following exclusion criteria: dental implants, crowding, and malocclusion. On average, each image includes six to eight teeth, and several patients presented teeth loss. The image resolution was 3200 × 2400.

### 2.2. Image Preprocessing and Teeth Detection (Region of Interest Definition)

The first step of the proposed method consists of an adaptive equalization operation (Figure 2b), performed to enhance the image’s details, allowing an easier differentiation between background and tooth areas. For this operation, we used the adaptive histogram equalization [21]. This operation considers parts of the image rather than the entire image. It uses the histograms of these parts to calculate local equalizations. Note that in the adaptive equalization, the histograms are created based on a defined neighborhood window. In this work, we tested variations of window sizes and achieved the best visual result using an 8 × 8 window.

In the achieved equalized image, the tonalities of teeth and background areas differ substantially. In this way, binary images can be obtained from the equalized images using a thresholding process. In this work, we used the Otsu thresholding technique [22].

Although the thresholding process properly separates most of the tooth areas from the background areas, some small regions are incorrectly assigned in both areas. This can be easily corrected using morphological operators [23]. Note that, in the binary image resultant from the thresholding (Figure 2c), the tooth areas are of large regions with few flaws. On the other hand, the incorrectly included regions are small and irregular, which can be eliminated easily using morphological operations. Considering the thresholded image (Figure 2c), we first applied an erosion using a structuring element that presents a rectangular shape and size of 130 × 20 (Figure 2d). We chose this specific element for erosion after evaluating the shapes of the incorrectly included regions. On the one hand, smaller elements do not eliminate the undesirable regions entirely; on the other hand, larger elements accidentally remove parts of the identified teeth regions. In our investigation, we also observed that using uniform, symmetrical structural elements, e.g., squares or circles, led to the union of regions of contiguous teeth that were close together. In the opening operation, a circle with a radius of 20 pixels was used as the structural element (Figure 2e). This operation eliminated the remaining undesirable parts. Finally, we applied dilation with a circle with a radius of 15 pixels as a structuring element. This dilation recovered the borders in the tooth areas that were accidentally removed in the erosion operation.

After removing the incorrectly included regions, the binary images are only composed of teeth areas (large and black) in a white background. In the resultant binary image, each area refers to a different tooth. These areas are considered the regions of interest. We created new images covering each tooth defined by the bounding boxes around the regions of interest (Figure 2f). Thus, we repeatedly cropped the original bitewing image, using the limits defined by the bounding boxes to obtain individual images for each tooth (Figure 2g). Data processing was performed using Python and the scikit-image library.

### 2.3. Data Labeling

As correctly pointed by Prados-Privado et al. [24], the histologic data can be considered the gold standard for caries diagnosis and is essential for the validation of caries diagnostic methods. Nevertheless, these data are very scarce. As discussed by Cehreli et al. [25], there is a lack of histologic data for several oral diseases. In the medical center in which this work’s data acquisition was performed, the histologic information was not available. This can be considered a limitation of this study, which is to be assessed in future works. In this way, the annotation of the images in this study was performed by experts based only on a visual evaluation of the radiographs.

The annotation of the 480 teeth images (defined by the regions of interest) were performed by an expert using the dataturks labeling tool (available at https://dataturks.com/, accessed on 3 March 2021 ). This process consisted of assigning a class (which can be normal, incipient, or advanced) to each detected tooth. The expert is an experienced dentist specialized in oral radiology. The labeling denoted that the set of 480 detected teeth is composed of 305 normal teeth, 113 teeth that present incipient lesions, and 62 teeth that present advanced lesions. This annotation was considered the ground truth in this study, i.e., the gold standard.

### 2.4. Dataset Split and Augmentation

We split the data into training and test sets. The training set is used to train the CNN models, and the test set is used in the models’ evaluation only. Note that the test set is not used in any part of the training process. For the test set, we separated 15 cases of each class, resulting in 45 teeth.

The remaining 435 tooth images (which consists of 290, 98, and 47 images for normal, incipient, and advanced classes, respectively) were submitted to a data augmentation process. To achieve a good performance in CNN algorithms, a reasonable volume of training data is essential [26]. However, in this study, as in the medical field in general, the number of images available for analysis is often highly restricted because it is difficult to obtain large amounts of similar data—even impracticable in some cases [27]. One alternative to consider in this context is the generation of synthetic data to increase the number of input samples. This process is called data augmentation.

Several operations can be applied to the original image set to generate new images. It is important to assure that the data augmentation process applied does not generate undesirable distortions in the image patterns. The data augmentation process used in our work consists of applying rotate and flip operations to the cropped teeth images. Considering that the dental arch structure shows high symmetry and that caries lesions can have a similar appearance when they affect teeth in any quadrant, we can use the flip operation to generate synthetic data. It simulates the occurrence of the same lesion in the equivalent teeth on the opposite side. The rotate operation simulates a slight variation in the acquisition of the image. In this operation, we used the following rotation angles: $-10^{\circ }$, $-5^{\circ }$, $5^{\circ }$ and $10^{\circ }$. The effects of all these operations are perfectly reasonable; therefore, they do not compromise the validity of our results.

After data augmentation, we obtained a total of 1160, 1176, and 1128 sample images for normal, incipient, and advanced classes, respectively. These sample images were used in the CNN’s training.

### 2.5. CNN Architectures

We selected the ResNet and Inception networks to be used in this work due to the promising performance presented by them in other medical image classification works [14]. The ResNet architecture is composed of several stacked ”Residual Units“, which are composed of two convolutional layers and two ReLU activation functions [20]. In total, it has 50 layers. The Inception network is composed of modules called “Inception modules” [19], which can be considered as individual networks; therefore, the entire architecture can be seen as a large architecture composed of smaller networks. Each Inception module is formed by 1 × 1, 3 × 3, and 5 × 5 convolutional layers and a 3 × 3 max pooling layer. We used the ResNet and Inception implementations provided by the Keras library.

### 2.6. Evaluation of Diagnostic Performance

In our evaluation of the CNNs models, we used the data in the test dataset and consider the following metrics: test accuracy, sensitivity (recall), specificity, positive predictive value (PPV, or precision), negative predictive value (NPV), and the area under the curve (AUC) of the receiver operating characteristic (ROC) curve. These statistical metrics are based on true negatives (correctly classified negative examples), true positives (correctly classified positive examples), false negatives (positive examples incorrectly classified as negative), and false positives (negative examples incorrectly classified as positive) [28].

Along with the statistical metrics, in which the CNNs’ results were compared with the ground truth, the results of the best CNN (according to the mentioned metrics) were also compared with the annotations of two dentists, who are familiar with oral radiology but have substantially less experience than the expert whose annotations were considered as ground truth (gold standard). For this last analysis, these two dentists evaluated the exams from which the cases that compose the test set were extracted. We separately asked them to point out, for each exam, the number of caries lesions of each stage (incipient and advanced). We registered this information for the mentioned analysis. Similarly, the results of the best CNN for the cases in the test set were also accounted per exam to be compared with the dentists’ findings. In total, 24 exams were evaluated in this last analysis, containing a total of 30 lesions: 15 incipient and 15 advanced, which are also part of the test set mentioned in the *Dataset split and augmentation* section. To verify if this number of exams can be used to provide an accurate statistical analysis, we calculated the minimum sample size using the R software. For that, we defined a test power of 95%, a significance level of 5%, and an effect size of 0.8. We obtained a value of 22 for the minimum sample size. Therefore, the number of examinations used in this last statistical analysis (24 exams) is enough to provide a valid evaluation.

The results gave by the best CNN and the less-experienced dentists were submitted to a paired Wilcoxon tests, considering as a variable the number of lesions for each stage per exam. In the first Wilcoxon test, the list of 24 values, referent to the number of incipient lesions found by the dentists per exam, was compared with the list of incipient lesions found by the best CNN. Similarly, in the second Wilcoxon test, the number of advanced lesions found per exam by the dentists and by the best CNN were compared. We considered a 95% confidence interval, i.e., a *p*-value > 0.05 would indicate that the hypothesis that the CNN and dentist’ results are statistically different is not valid, i.e., there is no statistical difference between them and, therefore, the results given by the method are equivalent to the results pointed by the dentists. The Wilcoxon test is a nonparametric statistical hypothesis test, used to evaluate data sets with unknown distributions. This test was also performed using the R software.

### 2.7. Experimental Setup

Table 1 presents the parameters used in the CNNs’ training. All models underwent a fine-tuning process, in which they were pretrained for 11,500 steps using the ImageNet dataset [29] to achieve better initial weight values. We used 20% of the training dataset in the validation step. Further, we varied the learning rate in three different values (0.1, 0.01, and 0.001) to evaluate which one would lead to better results. In that way, we analyzed six different CNN models: three ResNet models and three Inception models—one for each defined learning rate value.

The processes of training and testing of the CNNs were executed in a desktop machine with an Intel Xeon CPU 2.30 GHz processor (Intel, Mountain View, CA, USA), a Tesla P100-PCIE-16 GB GPU processor (Nvidia, Santa Clara, CA, USA), and 13 GB RAM.

### 2.8. Ethical Approval

All procedures performed in this study followed the ethical standards of the responsible committee on human experimentation (institutional and national) and the Helsinki Declaration of 1964 and later versions. The correspondent Research Ethics Committee approved the study (CAAE, registered at Brazilian Ministry of Health 24279314100005259). We confirm that all the methods applied observed the relevant guidelines and regulations.

## 3. Results

**Figure 3:**
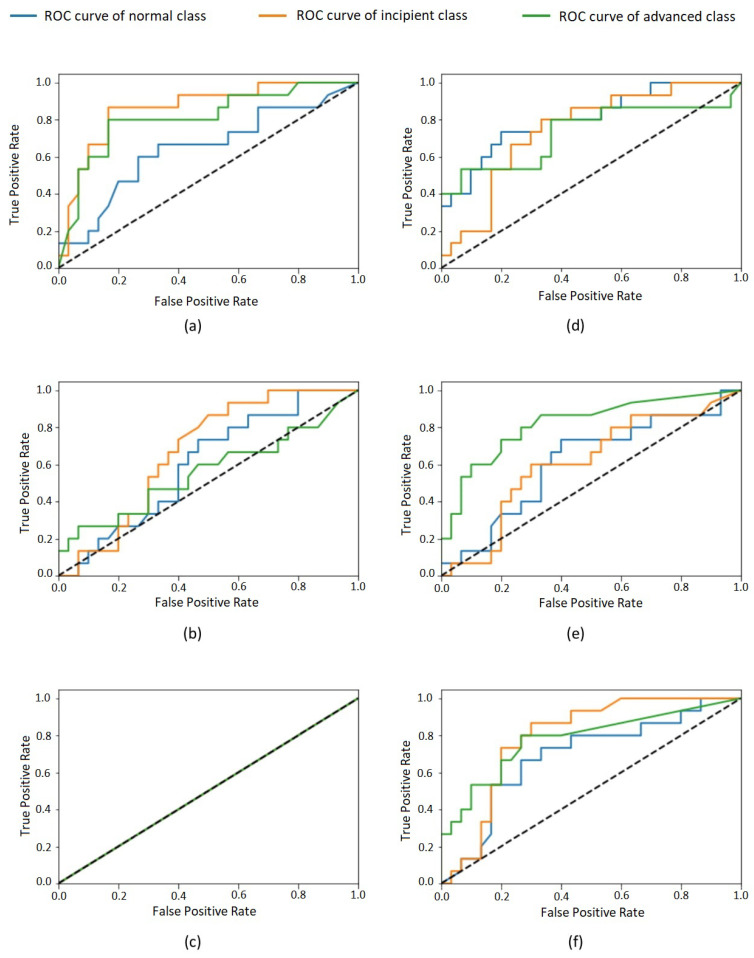
ROC curves of each class for model: Inception–learning rates (**a**) 0.001, (**b**) 0.01, and (**c**) 0.1; ResNet–learning rates (**d**) 0.001, (**e**) 0.01, and (**f**) 0.1. Normal class in blue, incipient in orange, and advanced in green.

After completing the training process, we evaluated six different CNN models using the test data set considering the following metrics: accuracy, sensitivity (recall), specificity, positive predictive value (PPV, or precision), negative predictive value (NPV), and the area under the curve (AUC) of the receiver operating characteristic (ROC) curve [30]. By evaluating the test data for each class, we obtained the values shown in Table 2. The overall results and the specific results for each class are summarized in the confusion matrices (see Table 3 and Table 4). Furthermore, the ROC curves for each class are shown in Figure 3.

In the last analysis, the best CNN’s results were compared with the findings of two less-experienced dentists. The CNN used in this last analysis was Inception, trained with the 0.001 learning rate since it presented the best results in the evaluation considering the test set’s ground truth (Table 2). There was no difference in the dentists’ annotations, i.e., not only did both less-experienced dentists present the same findings, but also these findings are in agreement with the ground truth (gold standard, expert annotations).

In that way, in the 24 exams from which the test cases were extracted, all dentists (expert and less-experienced dentists) found 15 incipient cases and 15 advanced cases. The best model (Inception trained with the 0.001 learning rate) found in the same 24 exams a total of 18 incipient lesions and 16 advanced lesions, as presented in Table 3. The *p*-value achieved by the Wilcoxon test for the hypothesis “The number of incipient lesions found by the dentists is different from the number of incipient lesions found by the best model” was 0.639. The Wilcoxon test for the hypothesis “The number of advanced lesions found by the dentists is different from the number of advanced lesions found by the best model” was 0.690. Note that for both Wilcoxon tests, the p-values achieved denoted that the hypotheses are not valid. Therefore, these results demonstrate that there is no statistical difference between the results presented by the less experienced dentists and the best CNN.

## 4. Discussion

Table 2 shows the performance of the evaluated models in the classification task considering the test set. For all models, a huge imparity in the performance for the three different classes is visible. This effect is also perceptible in confusion matrices (Table 3 and Table 4). The most-balanced results are obtained by the 0.001 Inception model. The remaining models presented very biased results—for example, the 0.1 Inception model, which classifies all test cases as belonging to the same class, as presented in Table 3. The other models disregard one or two of the classes, presenting a very low accuracy for them. Such bias phenomenon is also perceptible in the values in Table 2, which reflect a high volume of false positives and false negatives for some classes for most of the evaluated models. This imparity in the classification of different classes is also visible in the ROC curves of the classes (Figure 3) that, for most models, are very distant from one to another. It is possible to observe that none of the classes were unanimously favored by all models, so an increase in the whole dataset size would probably lead to better results.

Regarding the analysis presented here, the 0.001 Inception model presents the best results, considering the evaluation based on the test set, which suggests the feasibility of using the proposed method to classify approximal caries lesions in bitewing images.

The results achieved in the last analysis, based on the Wilcoxon test, suggest that the evaluated CNN presents a performance similar to the less-experienced dentists’ performance. All dentists indicate the same number of lesions for each exam considered. Their annotations are close to the CNN’s results, denoting a very similar number of incipient and advanced lesions. This similarity is also expressed in the high *p*-values, which confirms the hypotheses that the CNN’s results are statistically equivalent to the dentists’ results for the two stages. In that way, the results of this analysis suggest the feasibility of using CNNs as a decision support tool in the diagnosis of approximal caries.

Concerning the practical impact of the proposed method in a clinical setting, previous works have demonstrated the positive impact of adopting similar solutions in clinical routine [31]. In future steps, the solution proposed in this work will be integrated into a user interface so radiologists and dentists can use it to evaluate bitewing radiographs and detect caries lesions, and then include this analysis in their reports to be further considered in the treatment planning. Among the advantages of adopting the proposed method, one can mention the reduction in the interoperator diagnosis bias; the support in the detection of lesions that are not very visible for human experts; and the automatic definition of the lesion stages based on the tooth structures affected, which can lead to more adjusted treatments.

## 5. Conclusions

In this work, we propose and evaluate a method for classifying approximal caries in bitewing radiographs. We evaluate the use of two different CNN architectures in the classification task, varying their parameters, resulting in six different models. The best model presents promising results when compared to the ground truth using the traditional evaluation metrics and when compared with the performance of other dentists. Such results suggest that the proposed method can be used to assist dentists in the evaluation of bitewing images and approximal caries severity. As future works, in the next steps of this investigation, the histologic data will be included to be used as a gold standard method to corroborate the annotations and validate the proposed caries detection method.

## Figures and Tables

**Table 1:** Hyperparameters used in CNN training.

Parameter	Optimizer	Batch Size	Learning Rates
Value	Momentum	16	0.1, 0.01, and 0.001

**Table 2:** Performance of each CNN model considering the test set.

CNN	Learning Rate	Class	Precision	Recall	Specificity	NPV	AUC–ROC
Inception	0.001	Normal	0.818	0.600	0.933	0.823	0.643
		Incipient	0.722	0.866	0.833	0.926	0.861
		Advanced	0.687	0.733	0.833	0.862	0.810
	0.01	Normal	0.371	0.866	0.266	0.800	0.600
		Incipient	0.333	0.200	0.800	0.666	0.670
		Advanced	1.000	0.667	1.000	0.682	0.560
	0.1	Normal	0.000	0.000	1.000	0.667	0.500
		Incipient	0.000	0.000	1.000	0.667	0.500
		Advanced	0.333	1.000	0.000	0.000	0.500
ResNet	0.001	Normal	0.416	1.000	0.300	1.000	0.807
		Incipient	0.600	0.200	0.933	0.700	0.747
		Advanced	1.000	0.267	1.000	0.731	0.730
	0.01	Normal	0.379	0.733	0.400	0.750	0.612
		Incipient	0.333	0.200	0.800	0.667	0.609
		Advanced	0.714	0.333	0.933	0.737	0.819
	0.1	Normal	0.382	0.867	0.300	0.818	0.688
		Incipient	0.500	0.267	0.867	0.703	0.789
		Advanced	1.000	0.200	1.000	0.714	0.779

**Table 3:** Confusion matrices of each Inception model.

Learning Rate		Actual and Predicted Cases per Class			
0.001			PREDICTED		
			Normal	Incipient	Advanced
	TRUE	Normal	60%(9)	13%(2)	27%(4)
		Incipient	7%(1)	86%(13)	7%(1)
		Advanced	7%(1)	20%(3)	73%(11)
0.01			PREDICTED		
			Normal	Incipient	Advanced
	TRUE	Normal	87%(13)	13%(2)	0%(0)
		Incipient	80%(12)	20%(3)	0%(0)
		Advanced	67%(10)	27%(4)	7%(1)
0.1			PREDICTED		
			Normal	Incipient	Advanced
	TRUE	Normal	0%(0)	0%(0)	100%(15)
		Incipient	0%(0)	0%(0)	100%(15)
		Advanced	0%(0)	0%(0)	100%(15)

**Table 4:** Confusion matrices of each Resnet model.

Learning Rate		Actual and Predicted Cases per Class			
0.001			PREDICTED		
			Normal	Incipient	Advanced
	TRUE	Normal	100%(15)	0%(0)	0%(0)
		Incipient	80%(12)	20%(3)	0%(0)
		Advanced	60%(9)	13%(2)	27%(4)
0.01			PREDICTED		
			Normal	Incipient	Advanced
	TRUE	Normal	73%(11)	13%(2)	13%(2)
		Incipient	80%(12)	20%(3)	0%(0)
		Advanced	40%(6)	27%(4)	33%(5)
0.1			PREDICTED		
			Normal	Incipient	Advanced
	TRUE	Normal	87%(13)	13%(2)	0%(0)
		Incipient	73%(11)	27%(4)	0%(0)
		Advanced	67%(10)	13%(2)	20%(3)

## Data Availability

The data presented in this study are available on request from the corresponding author. The data are not publicly available due to privacy and ethical restrictions.
